# Expression of Class III Beta-Tubulin Is Associated with Invasive Potential and Poor Prognosis in Thyroid Carcinoma

**DOI:** 10.3390/jcm9123830

**Published:** 2020-11-26

**Authors:** Hee Young Na, Mira Park, Young A Kim, Jae Kyung Won, Young Joo Park, Sun Ah Shin, Sejoon Lee, Sohee Oh, Ji Eun Kim

**Affiliations:** 1 Department of Pathology, Seoul National University Bundang Hospital, Seoul National University College of Medicine, Seongnam 13620, Korea; 0614nina@hanmail.net; 2 Department of Pathology, Seoul National University Seoul Metropolitan Government Boramae Medical Center, Seoul National University College of Medicine, Seoul 07061, Korea; i01085790402@gmail.com (M.P.); pathgirl@daum.net (Y.A.K.); 3 Department of Pathology, Seoul National University Hospital, Seoul National University College of Medicine, Seoul 03080, Korea; jk_won@hanmail.net (J.K.W.); westps@hanmail.net (S.A.S.); 4 Department of Internal Medicine, Seoul National University Hospital, Seoul National University College of Medicine, Seoul 03080, Korea; yjparkmd@snu.ac.kr; 5 Precision Medicine Center, Seoul National University Bundang Hospital, Seongnam 13620, Korea; sejoonlee@snubh.org; 6 Department of Biostatistics, Seoul National University Seoul Metropolitan Government Boramae Medical Center, Seoul 07061, Korea; oh.sohee@gmail.com

**Keywords:** thyroid carcinoma, class III beta-tubulin, invasion, epithelial–mesenchymal transition, prognosis

## Abstract

Although American Thyroid Association guidelines offer a risk stratification scheme for thyroid cancer patients, there is a continuous need for more sophisticated biomarkers that can predict disease progression. In this study, we aim to evaluate the prognostic value of class III beta-tubulin (TUBB3) and uncover the relationship between TUBB3 and invasive potential in thyroid carcinoma. Immunohistochemistry (IHC) for TUBB3 and E-cadherin was performed on a total of 254 cases of thyroid cancer specimens. Tumor budding at the invasive margin was evaluated. In vitro functional studies were also performed; the protein and mRNA levels of TUBB3 were compared among the five cell types at baseline, with transwell invasion and after blocking of TUBB3 by shRNA. IHC revealed that the levels of TUBB3 were higher in conventional papillary carcinomas (cPTCs) and anaplastic thyroid carcinomas (ATCs). In univariate analysis, high tumor budding and TUBB3 expression were associated with inferior progression-free survival in cPTC. The results of a Western blot and RT-PCR agreed with the IHC finding. The results were further validated through data from The Cancer Genome Atlas database. Our results suggest that high expression of TUBB3 in thyroid carcinoma could predict invasive potential and possibly be linked with epithelial–mesenchymal transition.

## 1. Introduction

Thyroid cancer is one of the most common human malignancies with a steadily rising incidence worldwide. Due to the overall indolent behavior of this tumor, there have been controversies over managing all patients with radical treatment, including lymph node dissection [1,2]. Concerns about overdiagnosis and overtreatment have led to the concept of “active surveillance” in papillary microcarcinoma (PMC) [3,4,5]. However, despite a 5-year survival rate of nearly 100%, a minority of papillary carcinomas (PTCs), including aggressive histologic variants and even PMCs, may develop distant metastasis or local recurrence during the follow-up period, which severely affects patients’ quality of life [6,7,8,9,10]. This implies a biologic diversity of thyroid carcinoma, requiring more precise stratification to identify the subgroup that needs aggressive management and meticulous study. Because AJCC/TNM staging is focused on predicting the risk of death, various other systems such as EORTC, AGES, and AMES have been developed to assess the risk of recurrence [11,12,13,14], although no one system has shown superiority over the others. The 2015 American Thyroid Association (ATA) guidelines proposed an updated risk stratification system modified from the 3-tiered 2009 system, combining additional factors that include the extent of lymph node involvement and mutational status [15,16]. However, even this updated scheme requires further validation, and there is still an increasing demand for a new reliable biomarker.

Class III beta-tubulin (TUBB3) is one of nine beta-tubulin isoforms; it forms a heterodimer with alpha-tubulin and participates in building up microtubules, which play a role in maintaining cell shape, intracellular transport, and chromosome separation during mitosis and meiosis [17]. In epithelial tumors, TUBB3 has gained clinical significance as an indicator of resistance to Taxol-based chemotherapy [18,19,20,21]. Moreover, TUBB3 expression has been proven to be associated with aggressive features independent of chemotherapy status in various cancers, including breast, lung, and colon cancer [22,23,24,25,26,27,28]. Some researchers have suggested a relationship of TUBB3 with epithelial–mesenchymal transition (EMT), revealing a positive correlation between TUBB3 and some EMT markers, such as Snail [29]. This link has been more strongly suggested by Portyanko et al., who reported higher TUBB3 expression at the invasive margin and in tumor budding [26], and by Zhao et al., who demonstrated a positive correlation between TUBB3 and lymph node metastasis in colorectal carcinoma [28].

To date, there have been few studies exploring the role of TUBB3 in thyroid cancer [30,31,32,33]. In this study, we comprehensively investigated the expression of TUBB3 in various types of thyroid carcinomas, including the newly emerging entity “noninvasive follicular thyroid neoplasm with papillary-like nuclear features (NIFTP)”. We aim to evaluate the clinicopathologic significance of TUBB3 and its potential role as a therapeutic target in thyroid carcinoma.

## 2. Experimental Section

### 2.1. Patients and Samples

Samples of thyroid carcinoma were obtained from patients who received thyroidectomy at Seoul National University Hospital and Seoul National University Boramae Hospital from 1992 to 2013. The histopathologic review was confirmed by three experienced pathologists (H.Y.N., J.E.K., and J.K.W.), according to 2017 WHO Classification of Tumours of Endocrine Organs [34], including histologic types and degree of tumor budding, which is defined as the presence of isolated cells or clusters composed of fewer than 5 cells in the stroma at the invasive margin [35]. Clinical and pathological data were obtained from electronic medical records. This study was approved by the Institutional Review Board of Seoul National University Boramae Hospital (IRB No. 26-2017-44).

### 2.2. Immunohistochemistry (IHC)

Tissue microarray blocks were constructed with a pair of 3-mm-wide core tissues, including both tumoral and peritumoral stromal areas from the most representative paraffin-embedded blocks. For cases with small tumor volumes, such as PMC, whole section slides were used for IHC. IHC using anti-TUBB3 (TUJ1, 1:200; Covance, Princeton, NJ, USA), anti-E-cadherin (ECAD; HECD-1, 1:100; abcam, Cambridge, UK) antibodies, and BRAF-mutation-specific antibody (VE1, 1:100; Spring Bioscience, Pleasanton, CA, USA) was performed according to the validated protocols using an automated immunostainer (Ventana, Tuscon, AZ, USA). Of these, BRAF IHC was performed only in cPTC. The expression of TUBB3 was semiquantitatively estimated using an H-score modified from Levallet et al. [36]. The H-score was calculated by multiplying 3-tiered intensity scores (0, absent; 1, weak; 2, moderate to strong) and the frequency scores (the percentage of positive tumor cells). In the peritumoral stroma, TUBB3 expression was interpreted as low (absence or focal weak positivity) or high (moderate to strong positivity). For ECAD, the percentage of tumor cells showing loss of ECAD was manually calculated. Finally, a case was considered positive for BRAF VE1 if it displayed moderate to strong staining intensity, irrespective of the number of tumor cells stained [37].

### 2.3. Baseline Levels of TUBB3 in Thyroid Cell Lines

Human thyroid cell lines, including Htori-3 cells (normal thyroid follicular cells, kindly provided by Dr. YJ Park, Seoul National University Hospital), BCPAP cells (cPTC cells, a gift from Dr. YJ Park), MDA-T68 cells (follicular variant PTC (FVPTC) cells, purchased from ATCC, Manassas, VA, USA), BHT101 cells (anaplastic thyroid carcinoma (ATC) cells, provided by Dr. YJ Chae, Department of Surgery, Seoul National University Boramae hospital), and 8505C cells (ATC cells, a gift from Dr. YJ Chae), were used in the experiment. Protein expression of TUBB3 and ECAD was checked by Western blot and immunofluorescence (IF), and mRNA levels were investigated by reverse transcriptase PCR (RT-PCR). Western blotting was performed as previously described [38]. Briefly, a total of 30 μg of protein from each cell line was transferred to polyvinylidene difluoride membranes and incubated with primary antibodies against TUBB3 (TUJ1, 1:5000; BioLegend, San Diego, CA, USA), E-cadherin (HECD-1, 1:1000; Abcam, Cambridge, UK), vimentin (D21H3, 1:1000; Cell Signaling Technology, Beverly, MA, USA), and β-actin in TBST (TBS containing 0.05% Tween 20) at 4 °C overnight. After blocking and application of secondary antibodies, the bands were visualized using chemiluminescence. For IF, the cells were fixed with PBS containing 4% paraformaldehyde and incubated with the same primary antibodies, as used in the Western blot. A FITC IgG conjugate (Sigma Aldrich, St Louis, MO, USA) was used as the secondary antibody, and the nuclei were counterstained with 4′,6′-diamino-2-phenylindole (DAPI). RT-PCR was performed using TRIzol reagent (Thermo Fisher Scientific, Waltham, MA, USA) for RNA extraction and the QuantiTect Reverse Transcription Kit (Qiagen, Hilden, Germany) for cDNA construction. Amplification was conducted using the OneStep RT-PCR Kit (Qiagen). Specific primers targeting TUBB3 (amplifying a 353-bp fragment; forward, 5′-AAGCCGGGCATGAAGAAGT-3′ and reverse 5′-AGCAAGGTGCGTGAGGAGTA-3′) The amplified PCR products were resolved by 2% agarose gel and stained with ethidium bromide.

### 2.4. Transwell Invasive Assay

Transwell chambers (Corning Incorporated, Corning, NY, USA) were used for the invasion assay. Approximately 1 × 105 cells from each cell line were isolated before being added to the upper chamber of a transwell coated with Matrigel. After incubation for 24 h, the cells below the surface of the lower chamber were fixed with 4% paraformaldehyde solution and stained with Giemsa. The invading cells were observed in five different, randomly selected fields under a microscope. Expression of TUBB3 was visualized by immunofluorescence (IF) using the same primary antibody under a fluorescence microscope (Axio Imager Z1, Zeiss, Germany). Cells that penetrated the Matrigel were collected and examined for the levels of TUBB3, ECAD, and vimentin by Western blot and RT-PCR.

### 2.5. Blocking of TUBB3 by shRNA Transfection

Lentivirus expressing shRNA against TUBB3 (Santa Cruz Technology, Santa Cruz, CA, USA) was transfected into ATC cells (BHT101 and 8505C) using Lipofectamine 2000 (Thermo Fisher Scientific). ATC cell lines were cultured for 24 h, and 5 µg/mL shRNA was added to the cell lines. The cell lines without transfection were considered negative controls. Stable cell lines were selected with a medium containing 2 µg/mL puromycin for five more passages (12–15 days). The stable cell lines were harvested and validated with RT-PCR and Western blot for further experiments.

### 2.6. Utilization of Data from the Cancer Genome Atlas (TCGA)

We investigated the mRNA expression of TUBB3, ECAD, and vimentin as well as mutational profiles of TERT and BRAF genes in PTCs by using data extracted from cBioPortal for Cancer Genomics [39]. A total of 493 cases of PTCs, including 355 cPTCs, 102 FVPTCs (showing a follicular pattern in ≥99% of tumor), and 36 tall cell variant PTCs (tall cell component in ≥50% of tumor) with the standardized level 3 RNA sequencing data and clinical data were included in the analyses. To investigate the association of TUBB3 mRNA expression with EMT, we calculated an EMT score by subtracting the RNAseq by expectation-maximization (RSEM) values of 3 epithelial marker genes (CDH1, DSP, and OCLN) from the sum of RSEM values of 13 mesenchymal marker genes (VIM, CDH2, FOXC2, SNAI1, SNAI2, TWIST1, FN1, ITGB6, MMP2, MMP3, MMP9, SOX10, and GCS) [40]. The RSEM values of TUBB3 were log2 (+1) transformed before analyzing the differences among PTC variants.

### 2.7. Statistical Analysis

Spearman’s ρ was used to assess the correlation between TUBB3 expression and other clinicopathologic variables. The Mann–Whitney U-test and Fisher’s exact test were performed to compare TUBB3 expression and other clinicopathological parameters according to the subtypes of tumors. Overall survival was calculated from the date of diagnosis to the date of death. Progression-free survival was calculated from the date of diagnosis to the date of local recurrence or the date of lymph node or distal metastasis after the date of diagnosis. Univariate Kaplan–Meier survival analyses with log-rank tests were performed to compare binary groups based on TUBB3 expression levels. Multivariate survival analysis using the Cox multiple regression model was performed to identify independent prognostic markers. The results were considered statistically significant with a two-tailed *p*-value of 0.05. All data were analyzed with SPSS software, version 22.0 (SPSS Inc., IBM, Armonk, NY, USA).

## 3. Results

### 3.1. Patients

A total of 254 cases, including 123 cPTCs, 11 infiltrative FVPTCs, 84 invasive encapsulated FVPTCs (invEFVPTCs), 25 NIFTPs, and 11 ATCs, were enrolled in this study. The clinicopathologic parameters are summarized in Table 1. There were 53 male and 201 female patients, with a median age of 49 years (range 16 to 102). Regional lymph node metastasis at the time of diagnosis was found in 86 patients. During the follow-up period (median 170 months; range 0 to 300 months), disease progression, including death, local recurrence, and distant metastasis, was observed in 32 patients.

### 3.2. Differential Expression of TUBB3 and ECAD in Normal Thyroid Parenchyme and Thyroid Carcinomas

**Figure 1:**
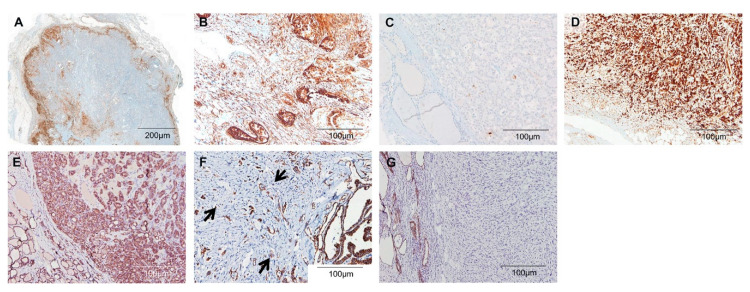
TUBB3 and E-cadherin expression in thyroid cancer. (**A**–**D**) TUBB3. (**A**,**B**) cPTC. (A) A low-power view of cPTC revealed TUBB3-positive tumor cells located at the invasive margin. (**B**) A high-power view showed strong TUBB3 expression in both stromal cells and tumor cells at the invasive front. (**C**) In EFVPTC, only a few scattered TUBB3-positive tumor cells were observed. Stromal cells were negative for TUBB3 in this case. (**D**) Tumor cells of ATC were strongly positive for TUBB3 in a diffuse pattern. (**E**–**G**) E-cadherin. (**E**) In cPTC, some tumor cells at the periphery showed loss of E-cadherin expression (arrows) while tumor cells at the center retained membranous E-cadherin expression. (**F**) Tumor cells in EFVPTC generally maintained membranous E-cadherin expression. (**G**) ATC tumor cells showed loss of E-cadherin expression throughout the tumor.

**Figure 2:**
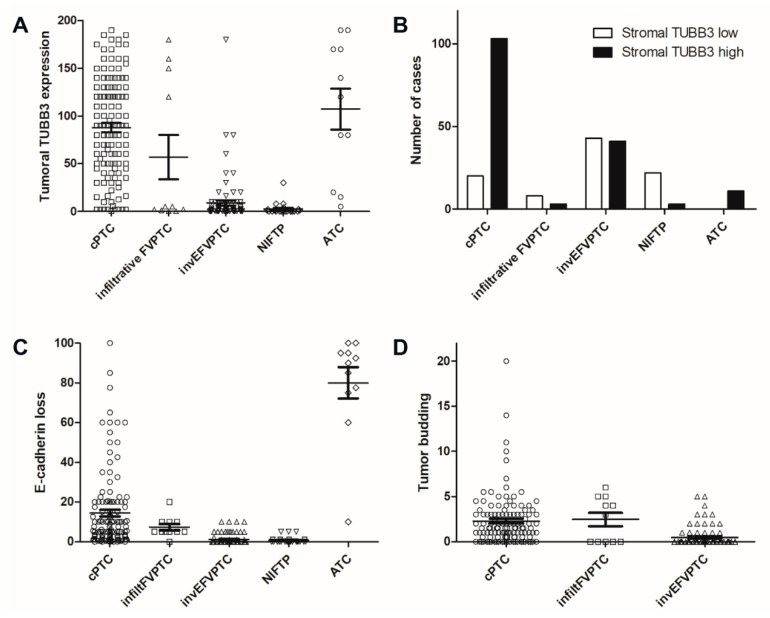
Differences of TUBB3 and E-cadherin expression and tumor budding among thyroid cancers. (**A**) TUBB3 H-score was highest in ATC, followed by cPTC, infiltrative FVPTC, invEFVPTC, and NIFTP. (**B**) High stromal TUBB3 expression was more common in cPTC and ATC than in invEFVPTC and NIFTP. (**C**) The proportion of tumor cells showing loss of E-cadherin expression, which was also highest in ATC, followed by cPTC, infiltrative FVPTC, invEFVPTC, and NIFTP. (**D**) Tumor budding was more commonly observed in cPTC and infiltrative FVPTC than in EFVPTC. cPTC; conventional papillary thyroid carcinoma, FVPTC; follicular variant papillary carcinoma, invEFVPTC; invasive encapsulated follicular variant papillary carcinoma, NIFTP; non-invasive follicular thyroid tumors with papillary-like nuclear features, ATC; anaplastic thyroid carcinoma.

In non-neoplastic thyroid tissues, both follicular cells and stromal cells were negative for TUBB3 and positive for ECAD in a diffuse membranous pattern. In thyroid carcinoma, TUBB3 and ECAD showed different expression patterns according to histologic subtypes (Table 1). Expression of TUBB3 was diffuse and strong throughout the tumor area in ATCs. In cPTCs and infiltrative FVPTCs, TUBB3 was selectively expressed in the tumor and stromal cells at the invasive margin, forming a band-like structure. However, invEFVPTCs and NIFTPs showed only a few scattered positive cells (Figure 1). ATCs revealed the highest levels of TUBB3, followed by cPTCs, infiltrative FVPTCs, invEFVPTCs and NIFTPs, both in tumor cells and stromal cells, although the differences between ATCs and cPTCs were not statistically significant. In contrast, the expression of ECAD showed the opposite pattern to that of TUBB3 (Figure 1 and Figure 2, Table 2 and Table S1).

**Figure 3:**
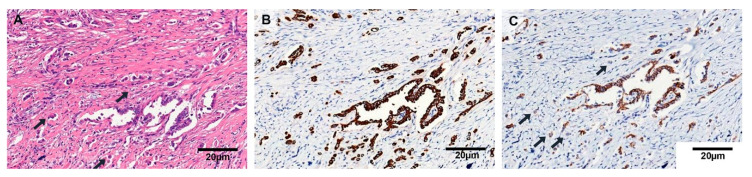
Tumor budding in cPTC. (**A**) A high magnification view showed cPTC with a single tumor cell or small clusters of tumor cells in fibrotic stroma at the periphery (×400). (**B**) These tumor buddings were positive for cytokeratin immunohistochemistry (×400). (**C**) Membranous E-cadherin expression was lost in some of these cells (×400).

Tumor budding showed strong TUBB3 expression and was frequent in cPTCs and infiltrative FVPTCs (Figure 3). In cPTCs, a total of 97 out of 123 cases (78.9%) had tumor budding, whereas 6 infiltrative FVPTCs (54.5%) and 17 invasive EFVPTCs (20.2%) showed budding. None of the NIFTP cases harbored these structures. Moreover, the number of tumor buddings was higher in cPTCs and infiltrative FVPTCs than in invEFVPTCs (all *p* < 0.05; Table 2 and Table S1).

**Figure 4:**
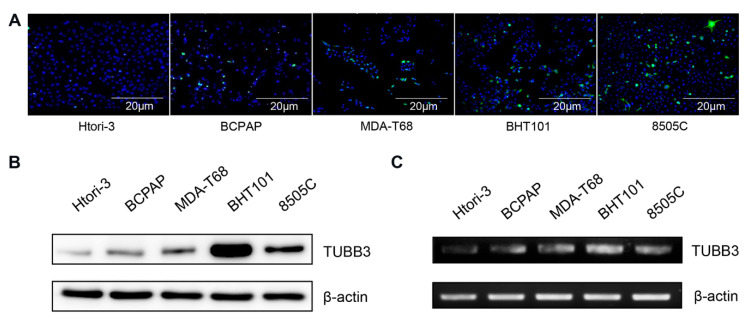
Western blot and RT-PCR in normal thyroid cells and thyroid cancer cells at baseline. (**A**) Immunofluorescent study revealed abundant TUBB3 (green) positive cells in ATC cells. TUBB3-positive cells showed spindle shape, suggestive of epithelial–mesenchymal transition. (**B**) Western blot and (**C**) RT-PCR revealed higher TUBB3 expression in ATC than in cPTC and FVPTC.

The results of Western blot and RT-PCR were concordant with those of IHC. The protein and mRNA expression of TUBB3 was highest in ATC cells (BHT101 and 8505C) and lowest in normal follicular cells (Htori-3). The cPTC (BCPAP) and FVPTC (MDA-T68) cells showed intermediate levels of protein and mRNA expression. An IF study revealed evident EMT-related morphological changes, including a loosening of cell contacts and the acquisition of a spindle shape, in TUBB3-positive cells (Figure 4) [41].

### 3.3. TUBB3 Was Upregulated during Stromal Invasion

**Figure 5:**
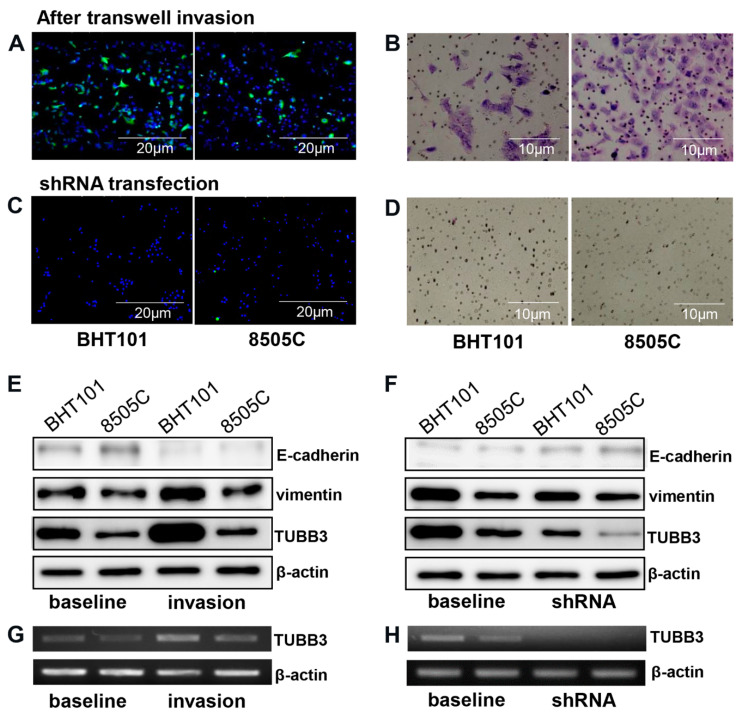
Transwell invasion assay and shRNA transfection. (**A**,**C**) Immunofluorescent and (**B**,**D**) Giemsa staining revealed reduced invasion activity of ATC cells after shRNA transfection. (**E**) Western blot revealed increased vimentin and TUBB3 expression and decreased E-cadherin expression after transwell invasion compared with baseline. (**G**) RT-PCR also revealed increased TUBB3 expression after transwell invasion. (**F**) Western blot after shRNA transfection showed decreased vimentin and TUBB3 expression and increased E-cadherin expression compared with baseline. (**H**) RT-PCR also revealed decreased TUBB3 expression after shRNA transfection.

After the transwell invasion assay, only ATC cells (BHT101 and 8505C) managed to permeate the Matrigel and successfully reached the bottom membrane. The mRNA levels of TUBB3 and vimentin were increased in the bottom chamber cells compared with the baseline levels, while the protein expression of ECAD decreased after the invasion. TUBB3 protein level also increased. These phenomena were reversely validated after shRNA-mediated downregulation of TUBB3 in ATC cells (BHT101 and 8505C). After transfection with TUBB3 shRNA, fewer cells invaded through the Matrigel, and both ATC cells showed significantly decreased vimentin but increased ECAD mRNA expression compared with cells at baseline (Figure 5).

### 3.4. Clinicopathological Correlation and Survival Analysis

In cPTCs, higher TUBB3 expression was correlated with stromal TUBB3 expression, higher tumor budding, and positivity for BRAF mutation-specific antibody (all *p* < 0.05). Additionally, high TUBB3 expression was associated with male gender, age over 55 years (all *p* < 0.05), and more frequent disease progression (*p* = 0.005). The loss of ECAD was associated with higher tumor budding and positivity for the BRAF mutation-specific antibody (all *p* < 0.05). High tumor budding was associated with BRAF positivity, age over 55 years, lymph node metastasis, advanced AJCC stage, and disease progression (all *p* < 0.05).

**Figure 6:**
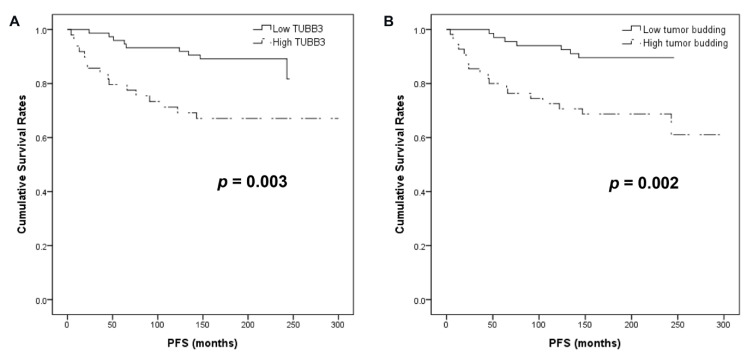
Kaplan–Meier analysis of patients based on TUBB3 H-score and tumor budding. (**A**) Higher TUBB3 H-score and (**B**) tumor budding were associated with shorter progression-free survival.

To evaluate the prognostic significance of various parameters, including TUBB3 expression, cases were divided into two groups based on the cut-off value calculated by a receiver operator characteristic (ROC) curve (the area under the ROC curve, 0.67). In univariate analysis, high TUBB3 H-score and tumor budding were associated with inferior progression-free survival (PFS; both *p* < 0.05; Figure 6). In multivariate analysis, distant metastasis (*p* = 0.002), high tumor budding (*p* = 0.004), and high TUBB3 H-score (*p* = 0.015) remained independent prognostic factors for inferior PFS. Meanwhile, only high stage (1 versus 2–4) remained an independent prognostic factor for inferior OS (*p* = 0.001; Table 3).

In invasive EFVPTCs, loss of ECAD was correlated with lymph node metastasis (*p* = 0.045). In infiltrative FVPTCs, NIFTPs, and ATCs, the expression of TUBB3 and ECAD did not show any association with clinicopathologic parameters.

### 3.5. TCGA Data Analysis

**Figure 7:**
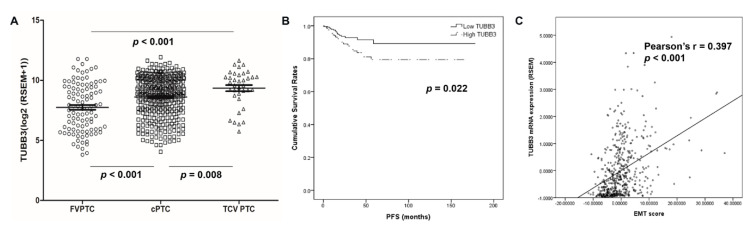
Validation in The Cancer Genome Atlas (TCGA) data. (**A**) TUBB3 mRNA expression was highest in tall cell variant PTC, followed by cPTC and FVPTC. (**B**) Patients with higher TUBB3 mRNA expression showed significantly shorter progression-free survival. (**C**) TUBB3 mRNA expression was correlated with a higher epithelial–mesenchymal transition (EMT) score.

The TUBB3 mRNA level was highest in tall cell variant PTCs (log2-transformed counts of 9.3 ± 1.5), followed by cPTCs (log2-transformed counts of 8.6 ± 1.7) and FVPTCs (log2-transformed counts of 7.7 ± 2.0; all *p* < 0.05; Figure 7A). The differences in ECAD and vimentin among the above variants were not statistically significant (Figure S1). However, higher TUBB3 mRNA expression correlated with a higher EMT score (Pearson’s r = 0.397, *p* < 0.001; Figure 7B). The correlation coefficients between 17 genes, including TUBB3 and 16 EMT-related genes, are shown in Figure S2. Additionally, higher TUBB3 mRNA expression was correlated with higher vimentin (*p* = 0.049) and the presence of BRAF V600E mutation (*p* < 0.001). The Kaplan–Meier survival analysis showed that patients with higher TUBB3 mRNA expression had shorter PFS than patients with lower TUBB3 mRNA expression (*p* = 0.041; Figure 7).

## 4. Discussion

We suggest that overexpression of TUBB3 can be induced during the stromal invasion of thyroid carcinoma and is an adverse prognostic marker. In our study, TUBB3 was expressed in tumor cells and the stroma of the invasive front, and upregulation of TUBB3 was observed during Matrigel penetration and was associated with an EMT phenotype. High TUBB3 expression, along with the presence of tumor budding, was a negative prognostic factor in cPTC. These results support that TUBB3 is a new biomarker predicting adverse outcomes in thyroid carcinoma.

A number of researchers have focused on TUBB3 because of its relationship with resistance to taxane-based chemotherapy [19,20,21]. To date, the relationship between TUBB3 and prognosis is not well defined. TUBB3 expression has been considered a predictor of tumor progression and aggressive behavior regardless of therapeutic options in various tumors [22,23,24,25,26,27,28]; however, other studies reported that high TUBB3 expression was associated with a superior response to drugs [42,43,44,45]. These findings indicate that TUBB3 expression might be regulated in a complicated pattern and influenced by various biological factors or microenvironments.

Very few attempts have been made to investigate TUBB3 expression and its prognostic impact in thyroid cancer, except for one study reporting the association between TUBB3 and taxane resistance in ATC [33]. More recently, Colato et al. demonstrated in their preliminary reports that TUBB3 expression was observed in the invasive margin of PTCs and metastatic foci in lymph nodes, while normal follicular cells, follicular adenomas, and encapsulated variant PTCs were negative for TUBB3 [30,31]. Similarly, the association between high TUBB3 expression and aggressive histologic features, including tall cell variant, squamous cell carcinoma or ATC components, angioinvasion, and disease recurrence, was revealed by Ciobanu et al. [32]. In line with these previous studies, TUBB3 expression was predominantly higher in ATC than PTC variants and normal thyroid follicular cells in our study. Validation of public data also consistently revealed higher TUBB3 expression in tall cell variant PTCs than cPTCs and FVPTCs. TUBB3 expression in cPTCs was associated with disease progression but not death in both our cohort and public data. This might be related to the overall indolent behavior of cPTC showing low frequency of death (8.1% in our cohort, and 4.2% in public data). Nevertheless, considering that AJCC/TNM staging is based on predicting death, not recurrence or progression [11], TUBB3 can be a useful marker associated with disease progression. Of note, TUBB3 was more frequently expressed in infiltrative FVPTCs than EFVPTCs or NIFTPs, which suggests that TUBB3 can be used as a diagnostic marker in these tumors with ambiguous histologic features.

In carcinoma, tumor cells at the invasive margin commonly undergo the EMT process, during which epithelial tumor cells lose polarity and cell–cell adhesion and acquire a mesenchymal phenotype [46]. EMT is considered critical for the initial steps of metastasis by enabling cells to gain motility and invasive potential [47], and the role of TUBB3 in the EMT process has yet to be uncovered. TUBB3 is a component of the microtubule that contributes to cell motility by generating protrusive force via interaction with actin filaments [48,49]. Additionally, recent studies have suggested that TUBB3 upregulates Snail and represses ECAD [29,50], and ubiquitination of TUBB3 is blocked by the long noncoding RNA RPPH1 during metastasis of colorectal cancer cells [50]. Our analyses of TCGA data also revealed a correlation between TUBB3 mRNA expression and EMT score, supporting previous studies. In our study, we observed EMT-related morphological changes, including tumor budding, in TUBB3-positive cells and found that migration and invasion were more active in thyroid cancer cells with high TUBB3 expression. Tumor budding is not only considered a morphologic presentation of EMT [51,52] but has also been validated as an important prognostic factor even in early-stage colon cancer [35,53,54]. Similar to these studies, we found that increased tumor budding was associated with advanced disease status and poor outcomes in cPTC. These results provide strong evidence and theoretical background for the claim that TUBB3 is activated through EMT and is a new prognostic biomarker. 

The mechanism of TUBB3 activation has not been clearly elucidated, and various biological factors can be hypothesized to play a role. Hypoxia [55] and poor nutritional status, such as hypoglycemia [56], have been suggested as important causes of enhanced TUBB3 expression [56]. These two phenomena are commonly seen in the peripheral areas of tumors and lead to genomic changes in cancer cells that can overcome the hostile microenvironment and induce cancer stem cell (CSC) characteristics [57]. Both hypoxia [58,59,60] and CSC features [61,62] are closely related to the EMT process. TUBB3 is considered a marker of CSCs because it is enriched in CSCs and induces multidrug resistance in coordination with ABC transporters [63,64]. These findings might explain why TUBB3 is selectively expressed in the peripheral part and invasive front of tumors and is associated with aggressive features in TUBB3-positive thyroid cancer.

In our study, TUBB3 was expressed not only in the tumor cells but also in the neighboring stroma. This suggests that microenvironmental factors that induce TUBB3 expression in tumor cells might also function in a similar way in stromal cells. Hypoxia contributes to the transformation of cancer-associated fibroblasts (CAFs), which encourage tumor growth and invasion to maintain stemness [57]. The source of TUBB3 in CAFs might be cancer cells, with paracrine regulation, and these CAFs are believed to participate in angiogenesis, tumor growth, and progression [65,66]. TUBB3 expression in thyroid carcinoma might occur with a tight connection with certain environmental stimuli and stromal interactions. 

High TUBB3 expression in thyroid cancer cells correlated with positivity to BRAF mutation-specific antibody. This finding coincides with the public data from cBioPortal, which revealed a correlation between TUBB3 mRNA expression and BRAF V600E mutation. However, considering that the great majority of cPTCs in Koreans harbor BRAF mutations [67] and virtually no BRAF mutations are found in NIFTPs or EFVPTCs [68], this might be an indirect result without causality.

## 5. Conclusions

In conclusion, the present study is so far the first to show the role of TUBB3 in the pathogenesis of thyroid cancer. Our study indicates that TUBB3 plays a role in tumor progression via EMT. TUBB3 could be a novel prognostic marker as well as a potential druggable target in PTC.

## Figures and Tables

**Table 1:** Clinicopathological parameters in different thyroid cancers.

Parameters	cPTC (*n* = 123)	Infiltrative FVPTC (*n* = 11)	invEFVPTC (*n* = 84)	NIFTP (*n* = 25)	ATC (*n* = 11)	Total (*n* = 254)
Sex						
Male, *n* (%)	21 (17.1)	0 (00.0)	20 (23.8)	9 (36.0)	3 (27.3)	53 (20.9)
Female, *n* (%)	102 (82.9)	11 (100.0)	64 (76.2)	16 (64.0)	8 (72.7)	201 (79.1)
Age (median, range) (years)	43 (16–102)	44 (24–63)	53 (26–73)	45 (26–71)	64 (54–77)	49 (16–102)
Tumor size (median, range)	2.1 (0.4–7.0)	2.0 (0.4–6.0)	1.7 (1.0–6.5)	1.9 (1.1–4.5)	4.0 (2.5–6.0)	2.0 (0.4–7.0)
Tumoral TUBB3 * (median, range)	90.0 (0.0–190.0)	5.0 (0.0–190.0)	1.0 (0.0–180.0)	1.0 (0.0–30.0)	120.0 (5.0–190.0)	21.0 (0.0–190.0)
Stromal TUBB3						
Low, *n* (%)	20 (16.3)	3 (27.3)	43 (51.2)	22 (88.0)	0 (0.0)	88 (34.6)
High, *n* (%)	103 (83.7)	8 (72.7)	41 (48.8)	3 (12.0)	11 (100.0)	166 (65.4)
E-cadherin loss (%, median, range)	6.0 (0.0–100.0)	5.0 (0.0–20.0)	0.0 (0.0–10.0)	0 (0.0–5.0)	90.0 (10.0–100.0)	0.8 (0.0–100.0)
Gross invasion, *n* (%)	25 (20.3)	0 (0.0)	0 (0.0)	0 (0.0)	3 (27.3)	28 (11.0)
Lymph node metastasis, *n* (%)	63 (51.2)	4 (36.4)	16 (19.0)	0 (0.0)	3 (27.3)	86 (33.9)
Distant metastasis, *n* (%)	4 (3.3)	1 (9.1)	0 (0.0)	0 (0.0)	5 (45.5)	10 (3.9)
Stage						
I, *n* (%)	88 (71.5)	9 (81.8)	73 (86.9)	23 (92.0)	0 (0.0)	193 (76.0)
II–IV, *n* (%)	35 (28.5)	2 (12.2)	11 (13.1)	2 (8,0)	11 (100.0)	61 (24.0)
Death, *n* (%)	10 (8.1)	0 (0.0)	0 (0.0)	0 (0.0)	4 (36.4)	14 (5.5)
Disease progression, *n* (%)	26 (21.1)	1 (9.1)	0 (0.0)	0 (0.0)	5 (45.5)	32 (12.6)
OS (months, median, range)	229 (4–300)	199 (3–273)	49 (0–261)	46 (1–170)	7 (2–18)	170 (0–300)
PFS (months, median, range)	219 (4–300)	199 (3–273)	49 (0–261)	46 (1–170)	5 (1–18)	88 (0–300)

* Tumoral TUBB3 in H-score. cPTC, conventional papillary thyroid carcinoma; FVPTC, follicular variant papillary thyroid carcinoma; invEFVPTC, invasive encapsulated follicular variant papillary thyroid carcinoma; NIFTP, noninvasive follicular thyroid neoplasm with papillary-like nuclear feature; ATC, anaplastic thyroid carcinoma; OS, overall survival; PFS, progression-free survival.

**Table 2:** Differential TUBB3 and E-cadherin expression among thyroid cancers.

Parameters	ATC vs. cPTC	ATC vs. Infiltrative FVPTC	ATC vs. invEFVPTC	ATC vs. NIFTP	cPTC vs. Infiltrative FVPTC	cPTC vs. invEFVPTC	cPTC vs. NIFTP
Tumoral TUBB3 †	0.293	0.04 *	<0.001 *	<0.001 *	0.065	<0.001 *	<0.001 *
Stromal TUBB3	0.369	0.214	0.001 *	<0.001 *	0.401	<0.001 *	<0.001 *
E-cadherin loss	<0.001 *	<0.001 *	<0.001 *	<0.001 *	0.702	<0.001 *	<0.001 *
Tumor budding	NA	NA	NA	NA	0.869	<0.001 *	<0.001 *

† Tumoral TUBB3 in H-score. * Statistically significant. ATC, anaplastic thyroid carcinoma; cPTC, conventional papillary thyroid carcinoma; FVPTC, follicular variant papillary thyroid carcinoma; invEFVPTC, invasive encapsulated follicular variant papillary thyroid carcinoma; NIFTP, noninvasive follicular thyroid neoplasm with papillary-like nuclear feature; NA, not applicable.

**Table 3:** Survival analysis in conventional papillary thyroid carcinoma.

Parameters	Univariate Analysis (OS)		Multivariate Analysis (OS)		Univariate Analysis (PFS)		Multivariate Analysis (PFS)	
	OR (95% CI)	*p*-Value	OR (95% CI)	*p*-Value	OR (95% CI)	*p*-Value	OR (95% CI)	*p*-Value
Age >55 years	3.182 (1.066–9.501)	0.038 *	-	NS	1.190 (0.587–2.409)	0.630		
Sex	0.650 (0.207–2.042)	0.461			0.450 (0.218–0.928)	0.031 *	-	NS
BRAF	0.790 (0.281–2.223)	0.655			0.668 (0.325–1.371)	0.271		
LN metastasis	0.616 (0.174–2.183)	0.453			1.686 (0.840–3.384)	0.142		
Distant metastasis	10.026 (2.121–47.386)	0.004 *	-	NS	4.536 (1.749–11.767)	0.002 *	4.939 (1.824–13.371)	0.002 *
Gross ETE	3.112 (1.107–8.744)	0.031 *	-	NS	1.046 (0.430–2.541)	0.921		
Stage (1 vs. 2–4)	7.925 (2.522–24.908)	<0.001 *	7.115 (2.230–22.702)	0.001	2.627 (1.311–5.262)	0.006 *	-	NS
Tumoral TUBB3 †	2.233 (0.746–6.682)	0.151			3.545 (1.743–7.210)	<0.001 *	2.690 (1.211–5.972)	0.015 *
Stromal TUBB3	1.490 (0.413–5.376)	0.543			2.276 (0.870–5.952)	0.094		
E-cadherin loss	0.588 (0.156–2.217)	0.433			1.696 (0.808–3.5559)	0.162		
Tumor budding	3.012 (0.779–11.650)	0.093			5.659 (2.528–12.672)	<0.001 *	3.903 (1.549–9.838)	0.004 *

† Tumoral TUBB3 in H-score. * Statistically significant. OS, overall survival; PFS, progression-free survival; OR, odds ratio; CI, confidence interval; NS, not statistically significant; ETE, extrathyroidal extension.

## Supplementary Materials

The following are available online at https://www.mdpi.com/2077-0383/9/12/3830/s1. Figure S1: Differences in expression of E-cadherin and vimentin among PTC; Figure S2: Correlation of mRNA expression level between TUBB3 and epithelial–mesenchymal transition (EMT)-related markers; Table S1: Differential TUBB3 and E-cadherin expression among thyroid cancers.
