# Supplementary material for: Expression of Class III Beta-Tubulin Is Associated with Invasive Potential and Poor Prognosis in Thyroid Carcinoma
Source: J Clin Med. 2020 Nov 26;9(12):3830. doi: 10.3390/jcm9123830 (PMC7760790; doi:10.3390/jcm9123830)
Supplement: Supplementary file 1 [file jcm-09-03830-s001.pdf]

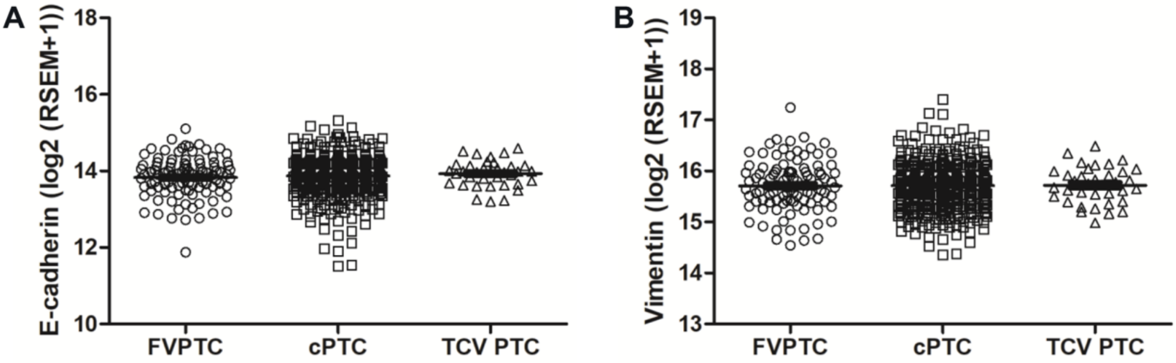

Figure S1. Differences in expression of E-cadherin and vimentin among PTC.

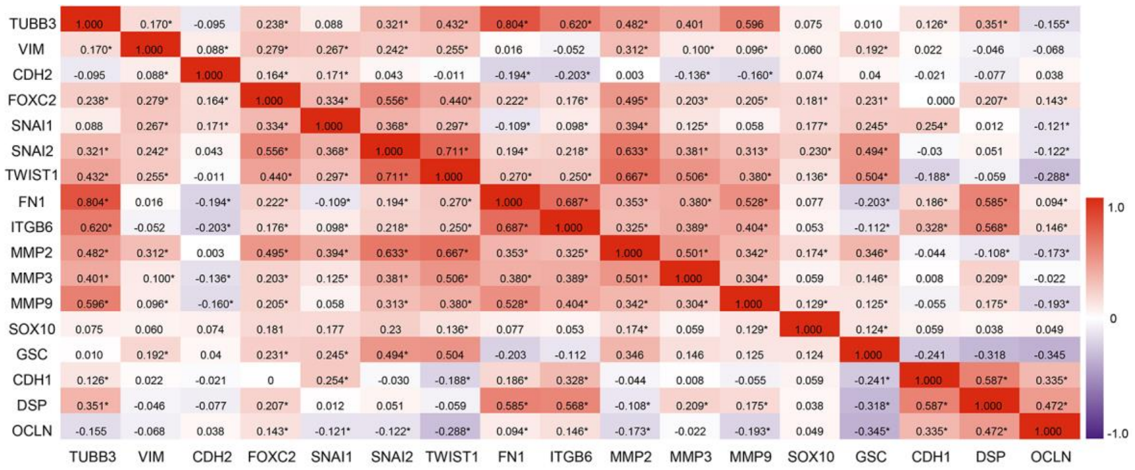

Figure S2. Correlation of mRNA expression level among TUBB3 and epithelial–mesenchymal transition (EMT)-related markers.

Table S1. Differential TUBB3 and E-cadherin expression among thyroid cancers.

Parameters	Infiltrative FVPTC vs invEFVPTC	Infiltrative FVPTC vs NIFTP	invEFVPTC vs NIFTP
Tumoral TUBB3	0.048*	0.001*	0.09
Stromal TUBB3	0.201	0.132	0.001*
E-cadherin loss	<0.001*	0.009*	0.214
Tumor budding	0.002*	0.001*	0.015*

*Statistically significant. FVPTC, follicular variant papillary thyroid carcinoma; invEFVPTC, invasive encapsulated follicular variant papillary thyroid carcinoma; NIFTP, non-invasive follicular thyroid neoplasm with papillary-like nuclear feature.
